# Magnetic Resonance Guided Radiation Therapy (MRgRT) Prostate Motion and Margins

**DOI:** 10.1002/jmrs.70033

**Published:** 2025-10-26

**Authors:** Sammi Peng, Tegan Courtot, Jessica Lye, Farshad Foroudi, Mark Tacey, Daryl Lim Joon, Michael Chao, Ee Siang Choong

**Affiliations:** ^1^ Department of Medical Imaging and Radiation Sciences, School of Primary and Allied Health Care Monash University Melbourne Australia; ^2^ Department of Radiation Oncology Olivia Newton‐John Cancer Wellness and Research Centre Heidelberg Victoria Australia

## Abstract

**Introduction:**

Systematic and random errors in radiation dose delivery necessitate the use of planning target volume (PTV) margins to ensure adequate clinical target volume (CTV) treatment. Advances in magnetic resonance‐guided radiation therapy (MRgRT) have enabled improved imaging with possible margin reduction; however, the optimal PTV margins remain uncertain. This study aimed to evaluate the adaptive radiotherapy component of intra‐fractional prostate movement in MRgRT for prostate cancer (PCa) patients and determine appropriate PTV margins.

**Methods:**

This study retrospectively analyzed 18 PCa patients treated using a 1.5 T MR‐Linac. The initial fusion MR and verification MR scans were registered offline to assess prostate displacement between the two scans in the anterior–posterior (AP), left‐right (LR) and superior‐inferior (SI) directions. Random and systematic errors were calculated, and the PTV margins were determined using the Van Herk formula.

**Results:**

The average time between MR scans was 22 min (range 9–54 min) compared to an average beam‐on time of 6 min (range 2–11 min). Mean and standard deviation of translational displacement was 1.2 ± 0.9 mm in the AP, 0.6 ± 0.5 mm in the LR, and 1.1 ± 0.8 mm in the SI directions. The calculated PTV margin was 3.2 mm in AP, 1.7 mm in LR, and 3.2 mm in SI directions. There was an observed trend of increased prostate motion with increased treatment duration.

**Conclusion:**

MRgRT facilitates PTV margin reduction for PCa; however, our findings suggest that increased on‐couch time may be associated with greater prostate motion. Future studies with larger patient cohorts and real‐time motion monitoring are recommended to optimise margin strategies.

## Graphical abstract

This study evaluated intra‐fractional prostate motion during MR‐guided radiotherapy to determine appropriate PTV margins for prostate cancer. Analysis of 18 patients resulted in calculated margins of 3.2 mm (AP), 1.7 mm (LR), and 3.2 mm (SI), supporting the potential for margin reduction with MRgRT.

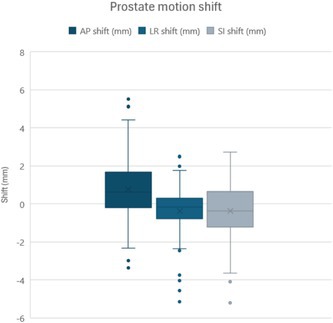

## 1 Introduction

Prostate cancer (PCa) is the second most common cancer among men worldwide [1]. Radiation therapy is an effective standard of care treatment option for many men with localized PCa [2], with proven favorable disease toxicity profiles and minimal impact on quality of life, particularly for low to intermediate‐risk PCa [3, 4]. The goal of radiation therapy is to deliver the prescribed dose to the tumor while sparing normal tissue [5], however, a number of systematic and random errors, including patient and prostate motion, contribute to an uncertainty in dose delivery to the clinical target volume (CTV). To mitigate these uncertainties, an added irradiated margin to create a planning target volume (PTV) is required to avoid a geographical underdosing of the target [6].

The introduction of image guidance in radiation therapy has allowed major advances in the precision of treatment delivery [7] associated with better definition of the tumour target, allowing reduction of the PTV margins and lower dose to surrounding organs at risk without compromising treatment to the CTV [8, 9]. A review of available literature has identified a range of PTV margins across different institutions depending on the image guidance technique used. Studies utilising alignment to skin marks or bony anatomy suggest that margins of 10 mm or more are required [10, 11], while use of fiducial markers or daily cross‐sectional imaging such as computed tomography‐guided radiation therapy (CTgRT) using cone‐beam CT (CBCT) allowed a reduction in margins to 5–8 mm [12].

Magnetic resonance‐guided radiation therapy (MRgRT) is a relatively new form of image‐guided radiation therapy (IGRT), enabling MRI scanning and radiation delivery on a single modality through the development of magnetic resonance imaging linear accelerators (MR‐Linacs) [13]. MR‐Linac systems have superior soft tissue visualisation, as well as real‐time tracking options on 2D cine‐MRI during delivery [14, 15], allowing better visualisation of radiotherapy targets and organs at risk than CBCT [16]. With such capabilities available to MRgRT, there is potential to reduce PTV margins and subsequently treatment toxicities [15]. The Elekta Unity MR‐Linac combines a 1.5 T MRI with a Unity 7 MV flattening‐filter‐free (FFF) Linac (Elekta Solutions AB, Stockholm, Sweden), allowing for online adaptive radiotherapy. This workflow involves an initial fusion planning MRI to which contours and dosimetry can be adapted to the daily position and to shape if required. The plan is recalculated and undergoes physics quality assurance checks before a second pre‐treatment verification MRI is performed to ensure no significant changes in position or shape while plan adaptation is being performed. This process is often lengthy, and thus, the prostate is susceptible to motion during this time.

The MRgRT system and daily adaptation can involve significantly greater overall time on the treatment couch than with CBCT. Previous literature has reported successful margin decreases to 2–3 mm with the use of MRgRT [17, 18, 19]. However, other studies have also demonstrated significant errors primarily attributed to prostate movement within the longer MRgRT workflow that suggest underdosage of the CTV and a risk of repeat planning when smaller margins are used [20, 21, 22]. In this study, we aimed to assess the inter‐MRgRT MRI and thus intrafractional motion variability of the prostate during the adaptation phases of 1.5 T MRgRT and estimate the required PTV margins.

## 2 Method

### 2.1 Study Population

The inclusion criteria for this study were patients with PCa and treated on Austin Health 1.5 T Elekta Unity MR Linear Accelerator between 1st October 2021 and 29th February 2024. Eighteen patients were retrospectively enrolled in the study. All eligible patients were required to have an intact prostate and treatment sites that required clinical imaging of the whole pelvis to ensure visibility of the entire prostate gland.

### 2.2 Treatment Planning and Delivery

Patient simulation using CT on a Siemens Somatom AS64 CT (Siemens, Erlangen, Germany) and MRI on Philips Ingenia Ambition 1.5 T MRI (Philips, Best, Netherlands) was completed for every patient. A T2 3D sequence with 1 mm slice thickness was used for the simulation MRI. Bladder and rectum control protocols were utilized for all simulation scans and treatment fractions, where patients were instructed to have an empty rectum and moderately full bladder of 300–500 mL by emptying the bladder and then drinking water 30 min prior to scanning. Barrigel rectal spacers were inserted prior to CT and MRI simulation where clinically indicated.

For the purposes of this study, conventionally fractionated (up to 20 fractions) and stereotactic (two to five fractions) PCa patients were included to provide sufficient data for statistical analysis. This included patients receiving treatment to the whole prostate for low‐risk patients, whole prostate ±1 cm of the seminal vesicles for intermediate‐risk cancer, and focal stereotactic treatments to an intraprostatic lesion, pelvic node/s, or seminal vesicle only. Images for all treatment fractions were assessed for prostate gland motion only, regardless of clinical treatment site, in line with the study protocol.

**FIGURE 1:**
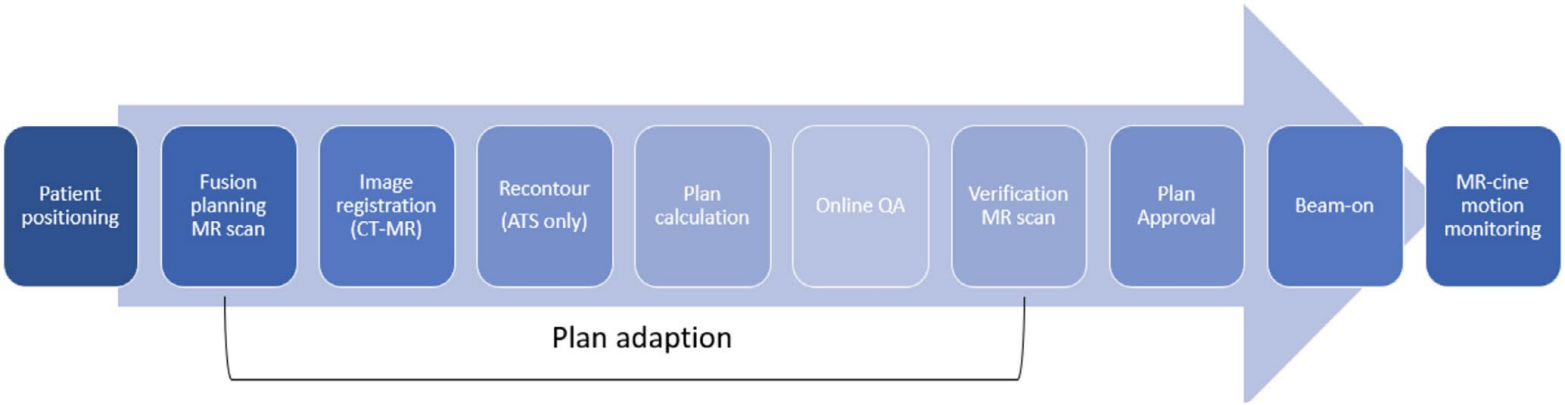
Schematic of workflow and the time frame where margins are assessed. ATS, adapt to shape; CT, computed tomography; MR, magnetic resonance; QA, quality assurance.

Treatment was performed on a 1.5 T Elekta Unity MR‐LInac. In each treatment fraction, as represented in Figure 1, a fusion planning MRI is acquired (2 or 4 min T2 3D), in which rigid registration is performed to the planning CT. Online adapt to position (ATP) is utilized for plan adaptation if required, and when ATP is not acceptable, adapt to shape (ATS) is completed before plan dosimetry is verified by the treating radiation oncologist and 2 radiation therapists, and the pre‐treatment plan quality assurance check is completed by a physicist. A verification MR (T2 3D) is then completed immediately prior to treatment to ensure no significant changes to anatomy. During treatment, intra‐fractional Cine‐mode motion monitoring is acquired, which can lead to treatment interruption or termination should the prostate move beyond the PTV margin.

The main source of uncertainty in this workflow is the residual patient movement during plan adaptation following the initial image‐guided correction from the daily fusion planning MRI. Plan adaptation can take from 10 to 50 min, and treatment margins need to include these changes. This can be quantified by comparison between the initial fusion planning image and the verification image taken immediately prior to treatment.

Although motion monitoring is used to interrupt treatment when the prostate exceeds the PTV margin, this data was not included in our analysis and thus an uncertainty component for motion during the treatment delivery should be included. In the BIR report [23], the uncertainty component for intra‐fraction motion can be included by using the tolerance on motion monitoring for gating, divided by √3. This assumes the target can be found anywhere within the stated tolerance with equal likelihood.

## 3 Data Analysis

Retrospective audit of radiation oncology and hospital electronic medical records (Mosaiq and Cerner) was completed to determine if patients met the inclusion criteria, and MR imaging for each fraction of treatment was retrieved from MIM Maestro (MIM Software, Cleveland, Ohio). Fraction zero (F0) treatment plans were included, and any days with a completion plan (where treatment was ceased based on motion monitoring and a second plan generated to complete treatment for that fraction) were entered as two separate fractions in the data set. Hence, some patients may have more MR‐Linac visits scheduled than the fractionation in the protocols mentioned above.

Offline rigid registration of the fusion planning and verification MR was completed using rigid fusion of the prostate gland between the two images, with displacement of the prostate gland only between the two images in left–right (LR), superior–inferior (SI), and anterior–posterior (AP) directions obtained. Organ rotation was set to a zero value and not included in the study, as the MR Linac only allows for translations in the three directions but not rotations. Patient shifts were defined as the difference in registration in each of the LR, SI, and AP directions.

The Van Herk formula [24] was utilized to calculate the margin required for the motion during plan adaptation.

$m_{\mathrm{ptv}}=\alpha \sum +\beta \sigma -{\beta \sigma }_{p}$

∑ represents the combined standard deviation of all systematic errors. This was calculated as the standard deviation of patient shifts and geometric distortion added in quadrature. The systematic patient shifts are calculated as the standard deviation of the individual patient mean shifts across their fractions. Geometric distortion was quantified using the physics 3Done geometric distortion phantom. This water‐filled phantom contains a grid with 2 cm spacing. The maximum prostate edge from isocentre for 10 patients was measured to be 2.4 cm A, 6 cm P, 2.5 cm L, 4.1 cm R, 4.7 cm S, and 7.7 cm I. The geometric distortion in the cardinal directions for points placed at the maximum extents of the prostate was determined, and the systematic error was assessed as the standard deviation in these points. A similar analysis was used by Nyholm et al. [25] although they assumed isotropic error.

*σ* represents the total standard deviation of all random errors. Random errors were calculated as the root‐mean‐square of the individual patient standard deviation shifts, inter‐observer difference, and penumbra width (*σ*_*p*_) of 3.2 as per Van Herk [24], added in quadrature.

Inter‐observer delineation variability was measured by comparing the AP, LR, and SI shifts for the MR image between two individuals for 5 fractions of different patients. The standard deviation in the difference in patient mean between the two individuals was calculated.

The value *α* = 2.5 was found by inspecting a 3D Gaussian probability density function for 90% confidence. The value of *β* = 1.64 for the 95% isodose surface, the distance between 50% and 95% dose levels of D_blurred_ (smeared planned dose distribution due to organ motion and setup error) as per Van Herk et al. [24].

The margins required for the motion during plan adaptation are then combined with the uncertainty estimate for residual motion during treatment delivery. The uncertainty component for intra‐fraction motion can be included by using the gating tolerance divided by √3, as random error [23]. The manual gating is triggered when the CTV exceeds the PTV contour, giving a gating tolerance equal to the PTV margin.

## 4 Results

### 4.1 Patient Characteristics

Table 1 summarises the characteristics of the study cohort. A total of 18 patients with favourable intermediate to high‐risk prostate cancer were included in the study. Patients were treated for several different treatment sites, including 11 patients treated for the whole prostate, 4 patients for intraprostatic lesions, 2 patients for positive pelvis nodes, and 1 patient for the left seminal vesicle. All patients underwent patient simulation with appropriate bladder and rectal preparation. One patient did not have a hydrogel rectal spacer inserted due to treatment for an intraprostatic lesion in the anterior prostate.

**TABLE 1:** Summary of patient characteristic.

Total participants (*n*)	18	
Variables	Median	Range
Age (years)	75	(49–82)
PSA (ng/mL)	8	(3.8–50)
Stage (Gleason score)	7	(6–9)
Number of fractions	5	(1–20)

Abbreviation: PSA, prostate specific antigen.

**FIGURE 2:**
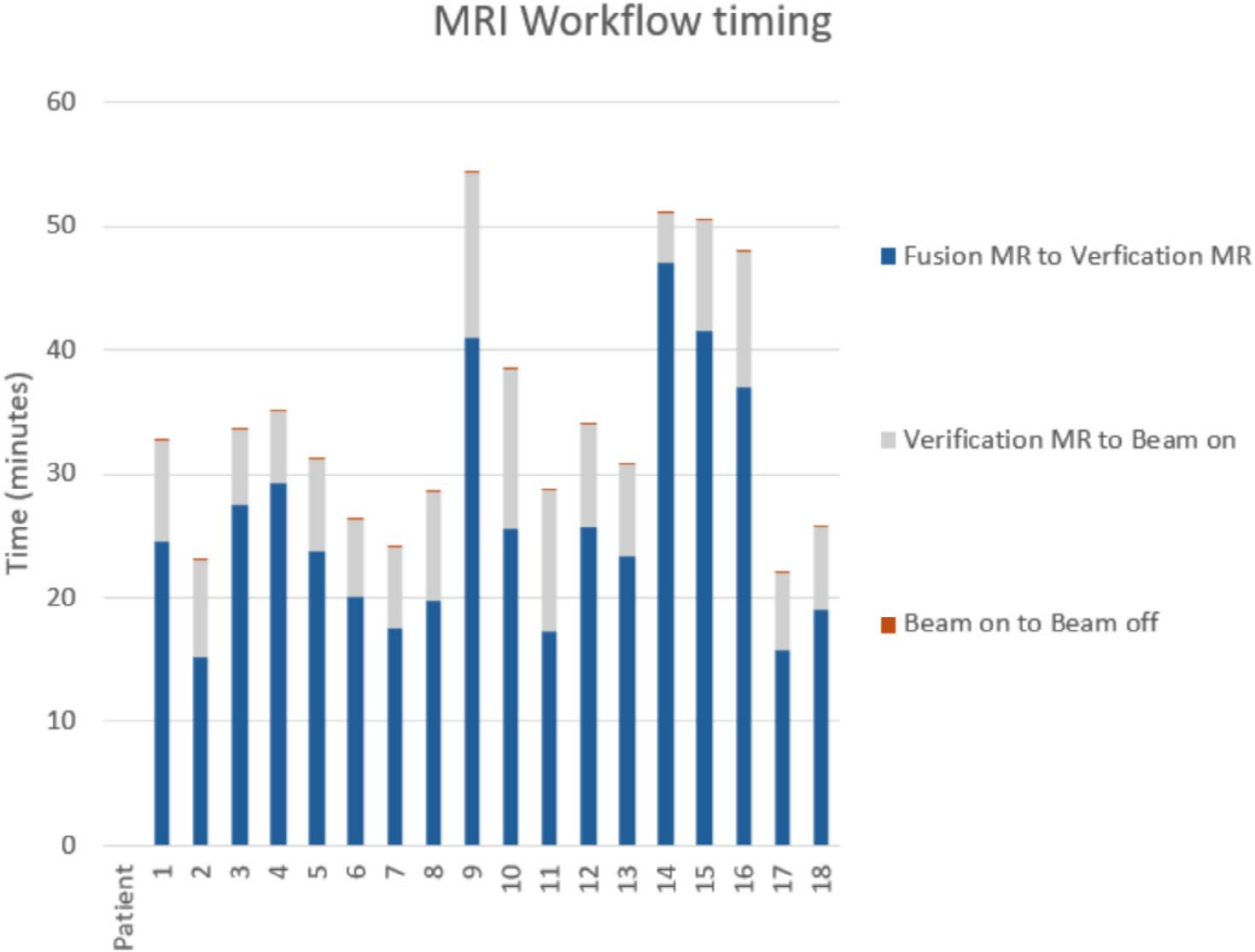
Graph representing average time spent in various phases of the MRgRT workflow for each of our 18 patients. MR, magnetic resonance.

Figure 2 demonstrates the average times of various phases of the MRgRT workflow for our patients. The average time from the beginning of the fusion MRI to beam‐off, representing the overall patient on‐table time, was 39 min (range 18–140). Beam‐on time was relatively short with an average time of 6 min (range 2–11 min). Time between fusion and verification MRI scans representing plan adaptation time contributed the majority of fraction time with an average of 22 min (range 9–54 min). This MR‐MR time was comparable between standard and stereotactic fractionation, where for stereotactic fractionation the average MR‐MR time was 25 min (range 10–54 min), and 22 min (range 9–54 min) for standard fractionation.

### 4.2 Prostate Motion During Plan Adaptation

A total of 170 fractions were analysed from the 18 patients. Table 2 shows the measured prostate shift and displacement in each of the AP, LR, and SI directions. The mean and standard deviation of prostate shifts were 0.8 ± 1.1 mm in the AP direction, −0.1 ± 0.6 mm in the LR direction, and −0.1 ± 1.1 mm in the SI direction. The mean and standard deviation of prostate displacement were 1.2 ± 1.1 mm in the AP direction, 0.6 ± 0.6 mm in the LR direction, and 1.1 ± 1.1 mm in the SI direction.

**TABLE 2:** Mean shift and displacement, standard deviation and RMS of prostate intra‐fractional motion.

	AP	LR	SI
Mean shift (mm)	0.8	−0.1	−0.1
Mean displacement (mm)	1.2	0.6	1.1
SD (mm)	1.1	0.6	1.1
RMS	1.1	0.7	1.0

Abbreviations: AP, anterior–posterior; LR, left–right; RMS, root mean square; SD, standard deviation; SI, superior–inferior.

**FIGURE 3:**
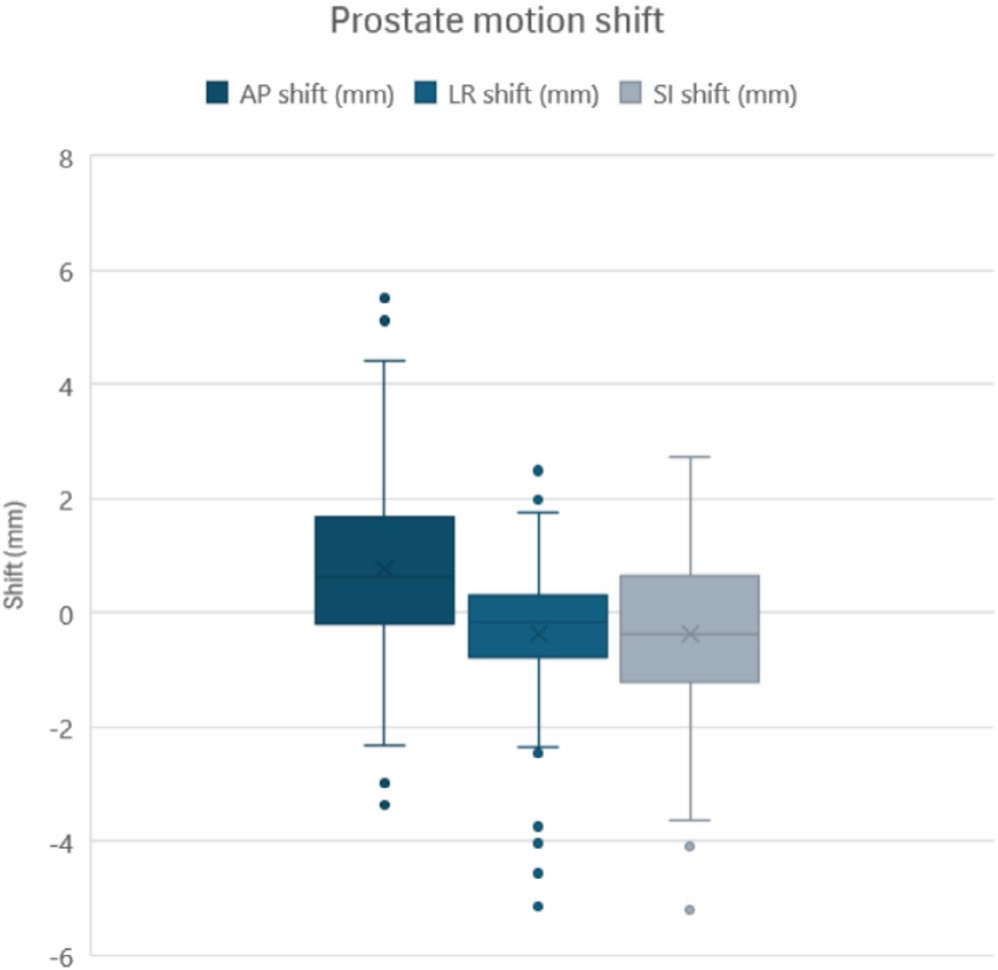
Prostate shift in the LR, AP, and SI directions. Positive values indicate anterior, left, and superior shifts, respectively, while negative values indicate posterior, right, and inferior shifts. AP, anterior–posterior; LR, left–right; SI, superior–inferior.

Figure 3 demonstrates the nature of prostate motion shift. Motion variation was greatest in the AP direction, with 7% of shifts greater than 3 mm in the AP direction, 5% in the LR direction, and 4% in the SI direction. 1% of shifts were greater than 5 mm in all directions. There was a slight systemic shift of 0.8 mm towards the anterior direction.

Inter‐observer delineation variability was measured between two individuals for five fractions, with a mean and standard deviation of 0.18 ± 0.25 mm in AP, 0.17 ± 0.24 mm in LR, and 0.35 ± 0.55 mm in SI directions.

### 4.3 PTV Margin

Table 3 outlines the systematic and random errors utilised to calculate the PTV margin during the plan adaptation phase. The PTV margin for which there is 95% of the nominal dose to the CTV for 90% of the patient population, as per Van Herk et al. [24] was calculated to be 3.2, 1.7, and 3.3 mm in the AP, LR, and SI directions, respectively. The group mean was 0.8, −0.1 and −0.1 mm in the AP, LR, and SI directions, respectively.

**TABLE 3:** Random and systematic error values utilized to calculate the PTV margin.

	AP	LR	SI
Systematic errors			
Prostate motion SD (mm)	1.12	0.59	1.14
Geometric distortion (mm)	0.25	0.14	0.31
Total systematic errors	1.15	0.61	1.18
Random errors			
RMS of shifts (mm)	1.09	0.71	0.96
Intra‐observer SD (mm)	0.25	0.24	0.55
Penumbra width (mm)	3.2	3.2	3.2
Total random errors	3.39	3.29	3.39
PTV Margin	3.2	1.7	3.3

Abbreviations: AP, anterior–posterior; LR, left–right; RMS, root mean square; SD, standard deviation; SI, superior–inferior.

Including an uncertainty component for intra‐fraction motion tolerance, taken as the above PTV margin/√(3), the margins increase to 3.9, 1.9, and 4.1 mm in the AP, LR, and SI directions, respectively.

### 4.4 Temporal Dependence

**FIGURE 4:**
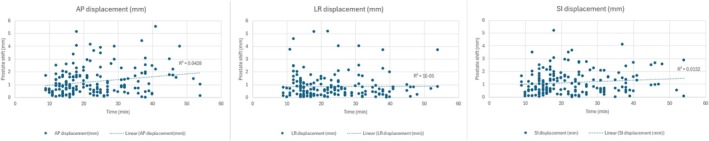
Graphs showing the relationship between prostate displacement and time between the fusion planning and verification MRI scan. AP, anterior–posterior; LR, left–right; SI, superior–inferior.

The relationship between prostate displacement and time between the fusion planning and verification MRI scans is shown in Figure 4. The average time between scans was 23 min. Linear regression analysis was completed, with an indication that AP displacement increased as time progressed; however, this was not statistically significant (*p* = 0.07).

## 5 Discussion

This study evaluated intrafraction motion of the prostate between fusion planning and verification MR images representing plan adaptation time in an MRL workflow, and the associated PTV margins. The mean and standard deviation of prostate shifts observed in our study were 0.8 ± 1.1 mm in the AP direction, −0.1 ± 0.6 mm in the LR direction, and −0.1 ± 1.1 mm in the SI direction. The greatest variation occurred in the AP direction, while the least occurred in the LR direction. These findings are consistent with existing literature, where LR has been consistently identified as having the least motion [20, 21, 26, 27], and AP and SI motion is comparable. Yang et al. [20] measured intra‐fractional motion between the two pre‐treatment scans in MRgRT, observing greatest motion in the AP direction. Kim et al. [21] measured MRgRT intra‐fractional motion using motion‐monitoring images and found greatest movement in the SI direction.

The PTV margin derived from our study was 3.2 mm in the AP direction, 1.7 mm in the LR direction, and 3.3 mm in the SI directions for whole and intraprostatic MR‐Linac‐guided prostate radiotherapy for the plan adaptation phase. These margins slightly increase when including uncertainty for the treatment delivery phase to 3.9, 1.9, and 4.1 mm for the AP, LR, and SI directions, respectively. These margins are smaller than the commonly used 5 mm margins for techniques using cross‐sectional imaging based on soft tissue registration [12], and are also smaller than the protocol currently used at our site for standard fractionation radiotherapy, which employs a 7 mm margin with 6 mm posterior. The margins calculated in this study are also smaller than the margins employed for MR‐guided stereotactic prostate radiotherapy at our site in the LR and SI directions, which employs 5 mm. In the AP direction, the stereotactic margins are similar to the margins calculated in this study, using 5 mm anterior and 3 mm posterior. The group mean in this study was 0.8 mm in the AP direction, which supports the use of asymmetric margins.

Studies evaluating intrafractional motion in MRgRT have indicated that 5 mm margins may be insufficient. Yang et al. [20] found minimum AP, LR, and SI PTV margins of 3.9, 2.8, and 5.3 mm for MRgRT when measuring the intrafractional motion using MR‐MR registration during both treatment adaptation and delivery phases, and margins of 2.9, 2.2, and 4.4 mm during just the plan adaptation phase. In the study by Yang et al. [20], motion is evaluated between the fusion planning and validation, and between validation and post‐treatment images, but no Cine‐mode motion monitoring occurs. For our analysis, with the improved accuracy afforded with motion monitoring, margins are established based on pre‐treatment motion, and it is assumed that treatment will be interrupted if these margins are exceeded. Mannerberg et al. [22] evaluated the dosimetric effects of 7, 5, and 3 mm PTV margins in the adaptation phase of an MRgRT workflow by comparing two MRI scans timed 30 min apart, with results indicating 2% and 4.2% undercoverage to the CTV for 5 and 3 mm margins respectively, attributed to significant bladder and rectal volume changes during the lengthy nature of the MRgRT workflow. Use of bladder and rectal protocols however should assist in mitigating motion due to surrounding organ changes, with position verification MRs and motion monitoring allowing confirmation of prostate position throughout the treatment fraction.

Prostate motion has been found to follow a random‐walk model constrained by anatomical boundaries [28], with an increase in motion over time [29, 30]. This is likely caused by changes in surrounding anatomy such as bladder volume and rectal filling [31]. In our study, we determined prostate motion between the fusion planning and the position verification MRI scans, representing adaptation time within an MRgRT workflow, which is a longer period compared to standard radiotherapy workflows. When evaluating the relationship between prostate displacement and time, positive trends were found in the AP and SI directions, suggesting that longer times contribute to greater prostate motion, though this relationship was not statistically significant.

Ideally, cine‐motion monitoring images and a post‐treatment MRI would be conducted and analyzed to better represent motion during the beam‐on treatment period each time, as the simplistic use of gating tolerance/√3 is conservative, and the actual residual motion during treatment is smaller. In our study, cine‐MR images acquired during beam‐on were not included in the analysis. Instead, the reported motion and subsequent PTV margins were based solely on prostate displacement detected between the pre‐treatment fusion and verification MRI images. Keizer et al. [28] evaluated prostate intrafractional motion during the entirety of an MRL fraction and identified that despite increases in motion over time, saturation of intrafractional motion appears to occur after approximately 30 min of on‐couch time. Our results show that adaptation contributes a significant portion of overall treatment time in comparison to beam‐on time, and other studies have also identified that prostate displacement during plan adaptation is more pronounced than during treatment delivery [20]. Westley et al. [32] monitored prostate motion during cine‐MR imaging, finding that 95% of patients spent 90% of time within 3 mm for AP and SI, and 100% of patients spent 90% of monitoring time within 2 mm for LR directions, consistent with the margins found in our study.

Other studies have supported the use of smaller margins. Dassen et al. [17] found isotropic 2.5 mm margins sufficient for online adaptive MRgRT, and Nikitas et al. [33] and Westly et al. [32] both concluded a 3 mm margin safe for MR‐guided SBRT. Additionally, Ma et al. [18] used 3 mm, and Kishan et al. [19] used 2 mm margins for MRgRT when comparing toxicity effects of CTgRT and MRgRT, finding decreased toxicity and good patient outcomes with MRgRT, attributed to the smaller margins. Our results support the use of smaller non‐isotropic margins for whole prostate MRgRT, particularly in the LR direction. It should be noted that in our study, motion analysis and subsequent PTV expansions were calculated for the prostate gland only; seminal vesicle motion was not assessed; therefore, reported margins are not applicable to the SVs. The slight systematic shift of the prostate in the anterior direction justifies the use of a larger PTV expansion anteriorly and smaller margins posteriorly. However, a larger study cohort may be required to confirm these findings.

Sources of intra‐fractional movement, in particular rectal and bladder filling, have been well documented in other studies [34], highlighting the importance of rectal and bladder protocols prior to treatment. In our study, hydro‐rectal spacers were implemented as part of a standard pre‐treatment protocol. There are known benefits in the use of rectal spacers in reducing rectal dose and toxicity [35]. The effects of rectal spacer insertion on prostate motion have been explored, where Cuccia et al. [36] found a significant difference in rotational shifts and an insignificant but positive trend for AP translational shifts between patients with and without rectal spacers. Yang et al. [20] also found a reduction in motion; however, also insignificant, when comparing patients treated for MRgRT with and without rectal spacers. Whilst results are not significant for translational motion mitigation, the continued use of rectal spacers is supported for both reduction in rectal dose and rotational motion.

Inter‐observer prostate delineation variability was minor in all directions for our study. This contrasts with other studies. Kim et al. [21] identified interobserver variability as a significant contributor to motion variation and thus margin calculations in MRgRT, with resulting differences of up to 0.7 mm in the calculated margin when excluding interobserver variability. Sanders et al. [37] also found significant interobserver variability when comparing delineation of the prostate between 7 observers on 50 MRI images. The small sample size in our study (five fractions assessed by two observers) limits the ability to draw inferences from our data.

The Elekta Unity MR Linac has also released Comprehensive Motion Management (CMM), which allows respiratory monitoring and tolerance gating. This allows real‐time tracking of the prostate during beam‐on, and a pause in radiation if it moves out of tolerance, as well as the possibility of intrafractional drift correction for systematic shifts without needing to cease treatment and re‐scan. The gating is configurable to interrupt treatment when the contour exceeds user‐defined volume, i.e., 95% of prostate volume exceeds CTV. This could allow even further margin reduction without significant increases in treatment time due to risk of insufficient CTV dose and should be a direction for future research.

Limitations of this paper include the exclusion of rotational shifts and deformations, as well as the small sample size and heterogeneity in the study sample. A focus on either standard or stereotactic fractionation could be investigated; however, given the similarities in time between the two in our studies, it is assumed that the margins can be applied to both. Additionally, evaluation of Cine‐motion monitoring and post‐treatment MR scans could allow better evaluation of intra‐fractional motion across the entirety of the MRgRT workflow. Furthermore, motion analysis in this study was limited to the prostate alone, with seminal vesicle motion not assessed. Future studies should also investigate seminal vesicle motion to determine whether different margins may be warranted.

## 6 Conclusion

In conclusion, intra‐fractional motion during adaptive MRgRT was measured using registration between fusion planning and verification MRI scans, and the associated PTV margins calculated using the Van Herk formula [24]. The high soft tissue contrast and better organ delineation, as well as motion monitoring capabilities in MRgRT, facilitate PTV margin reduction for prostate cancer. Current 5 mm with 3 mm posterior PTV margins for whole prostate only radiotherapy patients could be adjusted, particularly in the LR direction, to facilitate radiation dose reduction and toxicities. Future studies with larger patient cohorts and quantification of real‐time motion monitoring and CMM could be investigated to optimise margin strategies.

## Ethics Statement

This study and associated patient information sheets have been approved by the Austin Health Human Research and Ethics Committee (HREC). HREC Reference: HREC108024.

## Consent

As this study involved retrospective data, written informed consent was waived. No identifiable patient information was included in the manuscript.

## Conflicts of Interest

The authors declare no conflicts of interest.

## Data Availability

The data that support the findings of this study are available from the corresponding author upon reasonable request.
